# Association of adiposity and its changes over time with COVID-19 risk in older adults with overweight/obesity and metabolic syndrome: a longitudinal evaluation in the PREDIMED-Plus cohort

**DOI:** 10.1186/s12916-023-03079-z

**Published:** 2023-10-13

**Authors:** Sangeetha Shyam, Jesús Francisco García-Gavilán, Indira Paz-Graniel, José J. Gaforio, Miguel Ángel Martínez-González, Dolores Corella, J. Alfredo Martínez, Ángel M. Alonso-Gómez, Julia Wärnberg, Jesús Vioque, Dora Romaguera, José López-Miranda, Ramon Estruch, Francisco J. Tinahones, José Lapetra, J. Luís Serra-Majem, Aurora Bueno-Cavanillas, Josep A. Tur, Vicente Martín Sánchez, Xavier Pintó, Pilar Matía-Martín, Josep Vidal, Clotilde Vázquez, Lidia Daimiel, Emilio Ros, Fernando Fernandez-Aranda, Stephanie K. Nishi, Oscar Garcia-Regata, Estefania Toledo, Eva M. Asensio, Olga Castañer, Antonio Garcia-Rios, Laura Torres-Collado, Enrique Gómez-Gracia, M. Angeles Zulet, Nuria Goñi Ruiz, Rosa Casas, Naomi Cano-Ibáñez, Lucas Tojal-Sierra, A. M. Gómez-Perez, Jose V. Sorlí, Sergio Cinza-Sanjurjo, Sandra Martín-Peláez, Patricia J. Peña-Orihuela, Alejandro Oncina-Canovas, Rafael Perez-Araluce, María Dolores Zomeño, Alice Chaplin, Miguel Delgado-Rodríguez, Nancy Babio, Montserrat Fitó, Jordi Salas-Salvadó

**Affiliations:** 1 Centro de Investigación Biomédica en Red Fisiopatología de La Obesidad y La Nutrición (CIBEROBN), Institute of Health Carlos III, Madrid, Spain; 2 Universitat Rovira i Virgili, Departament de Bioquímica i Biotecnologia, Grup Alimentació, Nutrició, Desenvolupament i Salut Mental, Unitat de Nutrició Humana, Reus, Spain; 3 Institut d’Investigació Sanitària Pere Virgili (IISPV), Reus, Spain; 4 CIBER de Epidemiología y Salud Pública (CIBERESP), Instituto de Salud Carlos III (ISCIII), Madrid, Spain; 5 Departamento de Ciencias de La Salud, Instituto Universitario de Investigación en Olivar y Aceites de Oliva, Universidad de Jaén, Jaén, Spain; 6 Department of Preventive Medicine and Public Health, Instituto de Investigación Sanitaria de Navarra (IdiSNA), University of Navarra, Pamplona, Spain; 7 Department of Nutrition, Harvard T.H. Chan School of Public Health, Boston, MA USA; 8 Department of Preventive Medicine, University of Valencia, Valencia, Spain; 9 Department of Physiology and Nutrition, University of Navarra, Pamplona, Spain; 10 Precision Nutrition and Cardiometabolic Health Program, IMDEA Alimentacion, Madrid, Spain; 11 Medicine and Endocrinology, UVA, Valladolid, Spain; 12 Bioaraba Health Research Institute, Cardiovascular, Respiratory and Metabolic Area, Osakidetza Basque Health Service, Araba University Hospital, University of the Basque Country UPV/EHU, Vitoria-Gasteiz, Spain; 13 EpiPHAAN Research Group, School of Health Sciences, University of Málaga - Instituto de Investigación Biomédica en Málaga (IBIMA), Málaga, Spain; 14 Instituto de Investigación Sanitaria y Biomédica de Alicante, Universidad Miguel Hernández (ISABIAL-UMH), Alicante, Spain; 15 Health Research Institute of the Balearic Islands (IdISBa), Palma, Spain; 16 Department of Internal Medicine, Maimonides Biomedical Research Institute of Cordoba (IMIBIC), Reina Sofia University Hospital, University of Cordoba, Cordoba, Spain; 17 Department of Internal Medicine, Institut d’Investigacions Biomèdiques August Pi Sunyer (IDIBAPS), Hospital Clinic, University of Barcelona, Barcelona, Spain; 18 Institut de Recerca en Nutrició I Seguretat Alimentaria (INSA-UB), University of Barcelona, Barcelona, Spain; 19 Department of Endocrinology, Virgen de La Victoria Hospital, Instituto de Investigación Biomédica de Málaga (IBIMA), University of Málaga, Málaga, Spain; 20 Department of Family Medicine, Research Unit, Distrito Sanitario Atención Primaria Sevilla, Seville, Spain; 21 Research Institute of Biomedical and Health Sciences (IUIBS), University of Las Palmas de Gran Canaria & Centro Hospitalario Universitario Insular Materno Infantil (CHUIMI), Canarian Health Service, Las Palmas de Gran Canaria, Spain; 22 Department of Preventive Medicine and Public Health, University of Granada, Granada, Spain; 23 Research Group On Community Nutrition & Oxidative Stress, University of Balearic Islands, Palma, Spain; 24 Institute of Biomedicine (IBIOMED), University of León, León, Spain; 25 Lipids and Vascular Risk Unit, Internal Medicine, Hospital Universitario de Bellvitge-IDIBELL, Hospitalet de Llobregat, Barcelona, Spain; 26 Department of Endocrinology and Nutrition, Instituto de Investigación Sanitaria Hospital Clínico San Carlos (IdISSC), Madrid, Spain; 27 Medicine Department, Universidad Complutense, Madrid, Spain; 28 CIBER Diabetes y Enfermedades Metabólicas (CIBERDEM), Instituto de Salud Carlos III (ISCIII), Madrid, Spain; 29 Department of Endocrinology, Institut d`Investigacions Biomédiques August Pi Sunyer (IDIBAPS), Hospital Clinic, University of Barcelona, Barcelona, Spain; 30 Department of Endocrinology and Nutrition, Hospital Fundación Jimenez Díaz, Instituto de Investigaciones Biomédicas IISFJD, University Autonoma, Madrid, Spain; 31 Nutritional Control of the Epigenome Group, Precision Nutrition and Obesity Program, IMDEA Food, CEI UAM + CSIC, Madrid, Spain; 32 Departamento de Ciencias Farmacéuticas y de La Salud, Faculty de Farmacia, Universidad San Pablo-CEU, CEU Universities, Boadilla del Monte, Spain; 33 Lipid Clinic, Department of Endocrinology and Nutrition, Institut d’Investigacions Biomèdiques August Pi Sunyer (IDIBAPS), Hospital Clínic, Barcelona, Spain; 34 Psychoneurobiology of Eating and Addictive Behaviors Group, Institut d’Investigació Biomèdica de Bellvitge (IDIBELL), Barcelona, Spain; 35 Department of Clinical Psychology, University Hospital of Bellvitge and University of Barcelona, Barcelona, Spain; 36 Toronto 3D (Diet, Digestive Tract and Disease) Knowledge Synthesis and Clinical Trials Unit, Clinical Nutrition and Risk Factor Modification Centre, St. Michael’s Hospital, Unity Health Toronto, Toronto, ON Canada; 37 Unit of Cardiovascular Risk and Nutrition, Institut Hospital del Mar de Investigaciones Médicas Municipal d’Investigació Médica (IMIM), Barcelona, Spain; 38 Departament of Preventive Medicine, University of Málaga, Málaga, Spain; 39 Servicio Navarro de Salud-Osasumbidea, Pamplona, Navarra Spain; 40 Gerencia de Atención Primaria, Navarra, Spain; 41 IdiSNA, Navarra Institute for Health Research, Pamplona, Navarra Spain; 42 Instituto de Investigación Biosanitaria de Granada (Ibs.GRANADA), Granada, Spain; 43 CS MilladoiroÁrea Sanitaria de Santiago de Compostela, Santiago de Compostela, Spain; 44 Instituto de Investigación de Santiago de Compostela (IDIS), Santiago de Compostela, Spain; 45 Centro de Investigación Biomédica en Red-Enfermedades Cardiovasculares (CIBER-CV), Madrid, Spain; 46 School of Health Sciences, Universitat Ramon Llull, Barcelona, Spain; 47 Division of Preventive Medicine, Faculty of Medicine, University of Jaén, Jaén, Spain

**Keywords:** Obesity, COVID-19, Older adults, Weight loss, Central obesity

## Abstract

**Background:**

Cross-sectionally, older age and obesity are associated with increased coronavirus disease-2019 (COVID-19) risk. We assessed the longitudinal associations of baseline and changes in adiposity parameters with COVID-19 incidence in older adults at high cardiovascular risk.

**Methods:**

This analysis included 6874 men and women (aged 55–75 years) with overweight/obesity and metabolic syndrome in the PREDIMED-Plus lifestyle intervention trial for cardiovascular risk reduction. Body weight, body-mass-index (BMI), waist circumference, waist-to-height ratio (WHtR), and a body shape index (ABSI) were measured at baseline and annual follow-up visits. COVID-19 was ascertained by an independent Event Committee until 31 December 2021. Cox regression models were fitted to evaluate the risk of COVID-19 incidence based on baseline adiposity parameters measured 5–6 years before the pandemic and their changes at the visit prior to censoring.

**Results:**

At the time of censoring, 653 incident COVID-19 cases occurred. Higher baseline body weight, BMI, waist circumference, and WHtR were associated with increased COVID-19 risk. During the follow-up, every unit increase in body weight (HR_adj_ (95%CI): 1.01 (1.00, 1.03)) and BMI (HR_adj_: 1.04 (1.003, 1.08)) was associated with increased COVID-19 risk.

**Conclusions:**

In older adults with overweight/obesity, clinically significant weight loss may protect against COVID-19.

**Trial registration:**

This study is registered at the International Standard Randomized Controlled Trial (ISRCT; http://www.isrctn.com/ISRCTN89898870).

**Supplementary Information:**

The online version contains supplementary material available at 10.1186/s12916-023-03079-z.

## Background

Coronavirus disease-2019 (COVID-19) is a disease caused by severe acute respiratory syndrome coronavirus-2 (SARS-CoV2) infection, which emerged as a global pandemic towards the end of 2019 [1, 2]. SARS-CoV2 had a pandemic potential, unlike the previous zoonotic coronaviruses [3]. The unprecedented impact of the COVID-19 pandemic has had enormous consequences for global health and economy [4]. COVID-19 is known to have an extensive systemic health impact beyond affecting the respiratory system [5], with some of these consequences being persistent [6]. The public health restrictions to “flatten the spread” of the disease until vaccination [7] have also had enormous socio-economic implications [8]. Worryingly, climate change, increasing land use, urbanization, and global connectedness are likely to accelerate the emergence and transmission of novel zoonotic diseases [9]. Thus, it is important and urgent to understand the facilitators and barriers to disease transmission to be better prepared to prevent pandemics like COVID-19 and their catastrophic consequences from recurring.

Various modifiable and non-modifiable risk factors have been associated with higher susceptibility to severe COVID-19 and its complications. Prominently, several cross-sectional examinations from the early phase of the pandemic found that older adults and those with obesity were typically vulnerable to severe infection [10–13]. Longitudinal associations of adiposity changes are poorly documented in the literature. This evidence is important because while diet-induced weight loss has been shown to improve cardiovascular risks and innate immunity in younger patients with obesity [14], the benefits of weight loss have been debatable in older adults [15]. As the proportion of older adults increases globally [16], understanding long-term associations of adiposity parameters and their changes over time with the risk of zoonotic diseases such as COVID-19 in this target population could contribute to clinical management. Hence, we evaluated the longitudinal associations of adiposity parameters (body weight, body-mass-index (BMI), waist circumference, waist-to-height ratio (WHtR), and a body shape index (ABSI)) and their changes over time prior to incident infections with the risk of developing COVID-19 in older adults with metabolic syndrome. We performed this analysis within the PREvención con DIeta MEDiterránea Plus (PREDIMED-Plus) framework.

## Methods

### PREDIMED-Plus study

PREDIMED-Plus is a multicenter, randomized controlled trial in Spain assessing the effectiveness of an intensive lifestyle intervention on the primary prevention of cardiovascular diseases in comparison to usual care in 6874 community-dwelling older adults (women and men, aged 55–75 years). A detailed study protocol has been previously published [17, 18] and is available at https://www.predimedplus.com/. In brief, participants were eligible for study enrolment if they were overweight or obese (BMI between 27 and 40 kg/m^2^), and satisfied a minimum of three criteria for metabolic syndrome [19].

At enrolment, participants were free from cardiovascular disease and active cancer. The PREDIMED-Plus study hypothesizes that an intensive lifestyle intervention that encourages energy reduction with a high-quality Mediterranean dietary pattern and increased physical activity with motivational behavior support will have a larger reduction in the risk of hard cardiovascular events compared to usual care encouraging a Mediterranean diet [18]. The authors postulate that the greater risk reduction would result from the effectiveness of the intensive lifestyle intervention in facilitating long-term weight loss and maintenance, including reductions in waist circumference [18]. The 6-year trial intervention period has recently been completed and the in-person yearly follow-up for 2 years is currently ongoing and scheduled to be completed in 2024. The protocol of PREDIMED-Plus has approvals from the institutional review boards of all participating centers in line with the Declaration of Helsinki (Additional File 1_ SMethods for details). All enrolled participants provided written informed consent. This study is registered with the International Standard Randomized Controlled Trial Registry (ISRCT; http://www.isrctn.com/ISRCTN89898870).

The PREDIMED-Plus cohort has scheduled baseline and annual follow-up anthropometric, dietary, and physical activity data providing a cumulative assessment of the exposures before the COVID-19 pandemic. The documentation of sociodemographic and health data in the PREDIMED-Plus study also facilitates adjustments for potential confounders, making the study database well-suited to explore the prospective association of anthropometric parameters and their changes over time with the risk of COVID-19 incidence.

### Exposure: adiposity parameters

Adiposity parameters indicating general (body weight, BMI) and central (waist circumference and WHtR) obesity were assessed at baseline and yearly thereafter. This analysis assessed adiposity parameters from two perspectives: (i) values at baseline measured 5–6 years prior to the onset of the pandemic and (ii) changes from baseline over this time period (see below).

#### Baseline adiposity parameters

Body weight (kg), height (cm), and waist circumference (cm) of participants in light clothing were measured in duplicate by trained personnel at baseline and all annual visits. A mean of the duplicate measures for each visit was calculated. BMI was calculated as weight (kg)/height (m^2^). WHtR was calculated as a ratio of waist and height measurements in centimeters. A body shape index (ABSI), a measure of body shape independent of body weight and height, was calculated as waist circumference × weight^−2/3^ × height^5/6 ^[20]. ABSI was multiplied by 1000 to facilitate interpretation [21]. Participants were categorized into tertiles based on baseline body weight, waist circumference, WHtR, and ABSI. Participants’ BMI was categorized into overweight (BMI < 30kg/m^2^) or obesity (BMI ≥ 30kg/m^2^).

#### Changes in adiposity parameters

We calculated changes in adiposity occurring over two time points. Firstly, for the main analysis, “pre-censoring adiposity changes” for incident COVID-19 cases were defined as the difference in adiposity between the available value at the last visit before COVID-19 ascertainment and the baseline value. For non-incident COVID-19 participants, the last available adiposity data on or prior to the date of censoring (31 December 2021) or mortality was used. For a secondary analysis, we calculated adiposity changes from baseline until the last visit on/before 8 March 2020, when community transmission became widespread in Spain [22]. No incident COVID-19 cases were recorded in this cohort before this date. To facilitate clinical interpretation, participants were further categorized into three groups based on the percentage change in adiposity parameters: (i) those who experienced a gain, (ii) those who remained stable or achieved < 5% reduction, and (iii) those who achieved ≥ 5% reduction, relative to the baseline value. This categorization was undertaken since a weight loss of 5% is considered clinically significant [23].

### Outcome: COVID-19 incidence

A COVID-19 event was confirmed in a participant as adjudicated by the Clinical Event Ascertainment Committee of the PREDIMED-Plus trial based on medical records that were reviewed annually by physicians blinded to the intervention (see Supplementary Methods, Additional File 1_ SMethods). This analysis used only the first COVID-19 event in a participant adjudicated and confirmed from the start of the pandemic until 31 December 2021.

### Ascertainment of covariates

Sociodemographic data (age, sex, education level, marital status), health status, smoking habits, and alcohol consumption were self-reported by the participants at baseline and during the annual follow-up. The baseline data for these variables along with the participants’ recruitment center (location) were obtained for use as covariates.

In the PREDIMED-Plus, lifestyle information including adherence to an energy-reduced Mediterranean diet [24] and physical activity levels [25] was self-reported and documented using validated instruments for this population at all visits. Adherence to the energy-reduced Mediterranean was captured using a 17-item energy-restricted Mediterranean Adherence Screener (er-MEDAS) with a score range of 0–17 [24]. Higher er-MEDAS scores indicated higher adherence to the energy-restricted Mediterranean diet. Physical activity was assessed using the validated REGICOR questionnaire and total leisure-time physical activity-related energy expenditure was estimated in MET·min/week [26]. Baseline er-MEDAS and physical activity level data were obtained from the database for use as covariates.

Since exposure to angiotensin-converting enzyme (ACE) inhibitor drugs is known to affect COVID-19 risk [27], data on prior use of the medication until the pre-censoring visit was sourced from medical records. Data on whether the participants had obtained a first dose of a COVID-19 vaccine before censoring was also obtained from these records.

### Statistical analysis

The analysis included all 6874 randomized PREDIMED-Plus participants. A preliminary cross-sectional exploration was undertaken to compare participant characteristics across tertiles of baseline body weight and categories of body weight change at the pre-censoring visit. For this purpose, we used chi-square and Kruskal–Wallis tests, as appropriate. These results are described using median and interquartile range (IQR) or count and percentages for continuous and categorical data, respectively.

Because the exposures in this analysis were collected prior to outcome determination, we conducted a prospective analysis using the Cox proportional regression model, in which time to event for each participant began at randomization and ended at the time of COVID-19 diagnosis or the date of death or last contact on or prior to 31 December 2021, whichever occurred first.

The results of the main Cox proportional regression model using baseline adiposity parameters or their changes from baseline at the pre-censoring visit as the exposure, and COVID-19 status (incident case or not-incident) as the outcome, are presented as Hazards Ratio (HR) with 95% confidence interval (CI). In addition to the crude model without adjustments, two other models were tested. Model 1 was adjusted for baseline age (years), sex (male/female), education (primary or less/secondary/university), marital status (single or divorced/married/widow(er)), and recruitment center (location). Model 2 additionally adjusted for the intervention group, baseline smoking status (never/former/current), Mediterranean diet adherence score (17-point scale), total physical activity (METs.min/week), alcohol intake (g/d as a quadratic term), previous diagnosis of chronic diseases (diabetes, hypertension, hypercholesterolemia (Yes/No), prior use of ACE-inhibitor (Yes/No), and having received at least one dose of COVID-19 vaccine (Yes/No).

For modeling the linear association between absolute change in adiposity indicators (value at pre-censoring visit/value before 8 March 2020 − baseline value) and COVID-19 incidence, the respective baseline value was used as a covariate.

A simplified supplementary analysis was carried out comparing the HR in those who experienced any amount of weight gain to those who remained weight stable or lost any amount of weight. In this analysis, we used a third model to additionally adjust for the total numbers of leucocytes that have been implicated in inflammation and a positive diagnosis of COVID-19 [28]. Interactions of change in body weight with potential confounding factors (age group < 65 or ≥ 65 years, sex, smoking status, the prevalence of overweight/obesity, prevalence or absence of diabetes, hypercholesterolemia, and ACE inhibitor use) were assessed with a likelihood ratio test. Linear regression modeling stratified by these factors was undertaken and strata-wise HR for COVID-19 risk was inspected graphically.

An additional supplementary analysis using changes in adiposity parameters from baseline until 8 March 2020 (documented date of first community transmission of COVID-19 in Spain) as the exposure was performed using the same models as above to investigate their associations with COVID-19 incidence risk.

Additionally, sensitivity analyses were carried out to verify the results after excluding participants (*n* = 108) who had deceased prior to the known onset of the COVID-19 pandemic (i.e., death prior to 30 November 2019).

There were no missing data for age at trial entry, sex, education, intervention group, recruitment center, baseline physical activity, anthropometry, and prevalence of chronic conditions. Baseline smoking status and marital status had 0.4% missing data which were replaced with the mode of the variable for the cohort. Baseline alcohol consumption had 0.5% missing data, which was replaced with cohort mean consumption according to sex. Less than 0.1% of baseline Er MEDAS was missing and these were replaced with the cohort mean.

STATA (Version 14.2) was used to perform all analyses with the statistical significance set at 5%. PREDIMED-Plus database updated until 10 March 2023 was used for this analysis. All analyses were conducted with robust estimates of the variance to correct for intra-cluster correlation. Assuming that 10% of PREDIMED-Plus participants were diagnosed with COVID-19, the sample size of the trial provided 80% power to identify a 20% reduction in HR from one level of the exposure category in comparison to the other, assuming similar numbers in each category and with the statistical significance set at *p* < 0.05.

## Results

All 6874 participants randomized to this trial were available for the analysis, with 653 COVID-19-positive cases. Exposures were assessed over a median (IQR) follow-up of 5.8 (5.3–6.6) years which accounted for a total analysis time at risk of 40,497-person-years and an incidence rate of 16.1 (95%CI: 14.9, 17.4) per 1000 person-years.

At baseline, 5046 (73.4%) participants had obesity and the rest 1828 (26.7%) had overweight. Participant characteristics according to tertiles of baseline body weight are presented in Table 1. A heat map showing the correlations between the baseline adiposity indicators, stratified by sex, is presented in Supplementary Figure S1 (Additional File 2_Fig. S1). While BMI, height, and waist circumference showed high degrees of correlation with weight, ABSI, an indicator of body shape (central adiposity), showed lower degrees of correlation with general adiposity indicators such as body weight and BMI that are height dependent.

**Table 1:** Participant characteristics by tertile of baseline body weight

	Baseline body weight tertile			*P*-value^a^
	Tertile 1	Tertile 2	Tertile 3	
	(*n* = 2292)	(*n* = 2300)	(*n* = 2282)	
Sociodemographic data				
Age years ^#^^b^	66 (7)	65 (7)	63 (7)	< 0.001
Men, *n* (%)	463 (20.2)	1293 (56.2)	1783 (78.13)	< 0.001
Education level, *n* (%) ^b^				< 0.001
Primary school or less	1360 (59.3)	1101(47.9)	901 (39.5)	
High school or equivalent	566 (24.7)	661 (28.8)	759 (33.3)	
University	366 (16.0)	538 (23.4)	622 (27.3)	
Civil status, *n* (%)^b^				< 0.001
Single or divorced	302 (13.2)	298 (13.0)	302 (13.2)	
Married	1553 (67.8)	1724 (75.0)	1803 (79.0)	
Widow/Widower	437 (19.0)	278 (12.1)	177 (7.8)	
Intervention group (allocation to group B)	1118 (48.8)	1129 (49.1)	1159 (51.0)	0.342
Lifestyle habits				
Smoking habit, *n* (%)^b^				< 0.001
Never smoker	1382 (60.3)	958 (41.7)	694 (30.4)	
Former smoker	678 (29.6)	1043 (45.4)	1262 (55.3)	
Current smoker	232 (10.1)	299 (13.0)	326 (14.3)	
Study mean 17-item MedDiet score^1, b#^	9 (4)	8 (3)	8 (4)	0.009
Total physical activity, METs.min/week^b#^	1958 (2450)	1888 (2571)	1734 (2603)	0.079
Alcohol consumption, g/d ^b#^	2.2 (9.6)	5.8 (14.6)	8.8 (21.5)	0.098
Anthropometric and clinical data				
Weight (kg) ^#b^	73.8 (7.7)	85.6 (5.6)	99.2 (10.3)	< 0.001
Height (cm) ^#b^	155.0(9.0)	163.5 (11.5)	170.2 (10.1)	< 0.001
BMI, (kg/m^2^) ^#b^	29.9 (3.4)	32.0 (4.3)	35.0 (4.8)	< 0.001
Waist circumference (cm) ^#b^	99.4 (8.4)	107.0 (8.0)	116.0 (10.3)	< 0.001
Waist-to-height ratio^#^^b^	0.63 (0.06)	0.65 (0.07)	0.69 (0.08)	< 0.001
ABSI^#b^	82.4 (6.0)	83.1 (5.5)	83.5 (5.4)	< 0.001
Obesity; BMI ≥ 30, *n* (%)^b^	1131 (49.4)	1730 (75.2)	2185 (95.8)	< 0.001
Obesity; BMI ≥ 30, *n* (%) ^t^	989 (43.2)	1504 (65.4)	1993 (87.3)	< 0.001
Diabetes, n (%) ^b^	633 (27.6)	762 (31.3)	769 (33.7)	< 0.001
Hypercholesterolemia, *n* (%) ^b^	1711 (74.7)	1624 (70.6)	1478 (64.8)	< 0.001
Hypertension, n (%) ^b^	1901 (82.9)	1921 (83.6)	1936 (84.8)	0.204
Total, leucocytes (× 10^e9^/L)^# b^	6.46 (2.04)	6.58 (2.19)	6.79 (2.35)	< 0.001
Lymphocytes (× 10^e9^/L)^# b^	2.08 (0.90)	2.0 (0.91)	2.0 (0.94)	0.0105
Platelets (× 10^e9^/L)^# b^	233 (73)	222 (74)	211 (70)	< 0.001
Hemoglobin (g/dL)	14 (1.6)	14.5 (1.9)	14.8 (1.9)	< 0.001
Use of ACE inhibitors, *n* (%)^t^	825 (36.0)	859 (37.4)	875 (38.3)	0.257
Received at least 1 dose of vaccine, *n* (%)^t^	1203 (52.5)	1180 (51.3)	1120 (49.1)	0.065
COVID-19 incident cases, *n* (%)^t^	169 (7.4)	222 (9.7)	262 (11.5)	< 0.001
Time in trial at pre-censoring visit (years) ^#^	5.1 (1.0)	5.1 (1.0)	5.1 (1.0)	0.153
Body weight change (%)^tc^	− 2.2 (7.5)	− 2.2 (7.8)	− 2.6 (7.9)	0.039
Survival time (years) ^#^	5.8 (1.3)	5.8 (1.2)	5.8 (1.3)	0.061

*Abbreviations: ABSI* a body shape index, *ACE* angiotensin-converting enzyme, *BMI* body mass index, *COVID-19* coronavirus disease 2019, *MedDiet* Mediterranean diet

A body shape index (ABSI was calculated as waist circumference × weight^−2/3^ × height^5/6^. ABSI was multiplied by 1000 to facilitate interpretation

Data are *n* (%) for categorical variables. ^#^Unless specified, quantitative data are presented as median (IQR)

Age, sex, education, intervention group, recruitment center, baseline physical activity anthropometry, and prevalence of diabetes, hypertension, and hypercholesterolemia had no missing data for this analysis. Baseline smoking status: 28/6874 (0.4% missing data); marital status: 27/6874 (0.4%) missing data. Missing data for these two variables were replaced with the mode of the variable for the cohort. Alcohol consumption at baseline: 36/6784 (0.5%) missing data. Missing data was replaced with sex-specific cohort mean consumption (men = 17.47276; women = 4.599879 g/day). Mediterranean diet adherence: 5/6784 missing (< 0.1%) missing data. Missing data was replaced with cohort mean

^1^Notes on scales: Possible MedDiet scores range between 0 and 17. Higher MedDiet scores represent higher adherence to the Mediterranean diet

^a^*P*-values for comparisons between groups were tested using the Kruskal Wallis test (owing to the skewed nature of the distribution) or χ^2^, as appropriate

^b^Data from study baseline. ^t^Data at pre-censoring visit

^c^Body weight change refers to the difference in body weight between the value at the last visit prior to censoring and the baseline value

In preliminary comparisons, participants in the lowest tertile of body weight were older, more likely to be women, non-smokers, had lower levels of education, and more likely to be widowed at baseline. They had lower mean baseline BMI, waist circumference, WHtR, and ABSI than those in the higher body weight tertiles. They were also less likely to have diabetes, but more likely to have hypertension at baseline. They showed higher baseline adherence to the Mediterranean diet and were less likely to have tested COVID-19-positive during the follow-up. Those in the highest tertile of body weight at baseline had a higher total number of leucocytes compared to those in lower tertiles at the most recent visit prior to COVID-19, indicating higher levels of inflammation.

At the pre-censoring visit, 2260 (32.9%) participants had gained weight, 2432 (35.3%) remained weight stable or lost < 5% of their initial body weight, and 2182 (31.7%) lost ≥ 5% of their body weight relative to baseline. Participant characteristics according to body weight change category are presented at the pre-censoring visit in Supplementary Table S1 (Additional File 3_Table S1). Those who gained body weight were more likely to be current smokers at baseline. On the contrary, those achieving significant weight loss were also likely to have higher adiposity indices at baseline and presented a higher prevalence of diabetes and hypertension at baseline. Additionally, they were also more likely to have received at least one dose of the COVID-19 vaccine at the time of censoring. There was no significant difference in the duration spent in the trial at the pre-censoring visit between participants in varying body weight change categories.

Longitudinal associations between baseline adiposity indicators and the risk of COVID-19 incidence are presented in Table 2. All baseline adiposity indicators showed positive longitudinal associations with COVID-19 risk, even when adjusted for potential confounders including sex and vaccination status. When evaluated as tertiles, those in the highest tertile of body weight (HR (95%CI): 1.46 (1.17, 1.83)) and WHtR (HR:1.22 (1.01,1.47)) had significantly higher risks compared to those in the respective lowest tertile. Having obesity versus having overweight at baseline, significantly increased the risk of COVID-19 by an average of 27% (95%CI: 5 to 53%), when fully adjusted. Every additional centimeter in baseline waist circumference was associated with a 1% increase in COVID-19 risk (95%CI: 0.4 to 2% increase) in the fully adjusted model. Body shape indicator (ABSI) was not associated with COVID-19.

**Table 2:** Baseline adiposity measurements and risk of COVID-19 (HR and 95%CI)

	No. of cases/total	Crude Model	Model 1	Model 2
Body weight				
Tertile 1	169/2292	1 (ref)	1 (ref)	1 (ref)
Tertile 2	222/2300	1.34 (1.09, 1.63) **	1.27 (1.03, 1.56) *	1.26 (1.02, 1.55) *
Tertile 3	262/2282	1.62 (1.34, 1.96) ***	1.48(1.19, 1.85) ***	1.46 (1.17, 1.83) ***
Linear (per 1kg increase)	653/6874	1.01 (1.01, 1.02) ***	1.01 (1.005, 1.02) ***	1.01 (1.004, 1.02) ***
Body mass index (BMI)				
Category: overweight	145/1828	1 (ref)	1 (ref)	1 (ref)
Category: obesity	508/5046	1.27 (1.06, 1.54) **	1.29 (1.07, 1.56) **	1.27 (1.05, 1.53) *
BMI linear (per 1kg/m^2^ increase)	653/6874	1.04 (1.02, 1.06) ***	1.04 (1.02, 1.06) ***	1.04 (1.02, 1.06) ***
Waist circumference				
Tertile 1	185/2361	1 (ref)	1 (ref)	1 (ref)
Tertile 2	229/2224	1.32 (1.09, 1.61) **	1.27 (1.04, 1.56) *	1.20 (0.98, 1.48)
Tertile 3	239/2289	1.38 (1.13, 1.67) ***	1.30 (1.06, 1.59) *	1.22 (0.99, 1.51)
Linear (per 1cm increase)	653/6874	1.02 (1.01, 1.02) ***	1.01 (1.004, 1.02) **	1.01 (1.002, 1.02) *
Waist-to-height ratio				
Tertile 1	225/2293	1 (ref)	1 (ref)	1 (ref)
Tertile 2	175/2290	0.77 (0.63, 0.93) **	0.80 (0.66, 0.98) *	0.79 (0.64, 0.96) *
Tertile 3	253/2291	1.16 (0.97, 1.39)	1.24 (1.04, 1.49) *	1.22 (1.01, 1.47) *
Linear (per 0.03-unit increase)	653/6874	1.04 (1.00, 1.08)	1.06 (1.01, 1.10) **	1.05 (1.01, 1.10) *
ABSI				
Tertile 1	224/2292	1 (ref)	1 (ref)	1 (ref)
Tertile 2	220/2291	0.99 (0.82, 1.19)	0.94 (0.77, 1.13)	0.92 (0.76, 1.12)
Tertile 3	209/2291	0.95 (0.79, 1.15)	0.91 (0.74, 1.12)	0.88 (0.71, 1.08)
Linear (*per m*^*11/6*^* kg*^*−2/3*^*unit increase*)	653/6874	1.00 (0.98, 1.02)	1.00 (0.98, 1.02)	0.99 (0.97, 1.01)

HR (95% CI) was calculated using Cox Proportional regression models. Exposure = baseline anthropometric data; outcome: Covid-19 incidence (Y/N)

Tertile 1 had the lowest value and tertile 3 had the highest value

A body shape index (ABSI) was calculated as waist circumference × weight^−2/3^ × height^5/6^. ABSI was multiplied by 1000 to facilitate interpretation

The crude model used no adjustments

Model 1: Adjusted for baseline age (years), sex (male/female), education (primary or less/secondary/university), marital status (single or divorced/married/widow(er), and recruitment center

Model 2: Additionally, adjusted for baseline smoking status (never/former/current), intervention group, Mediterranean diet adherence score (17-point scale), total physical activity (METs.min./week), alcohol intake (g/d as a quadratic term), and diagnosis of chronic diseases (diabetes, hypertension, hypercholesterolemia (Y/N)), use of ace-inhibitor at/before pre-censoring visit (Y/N), and having one dose of COVID-19 vaccine at the time of censoring (Y/N)

For waist-to-height ratio change, linear association with COVID-19 is calculated per 0.03-unit increase which approximately denotes a 5% increase from the average value for this cohort

^*^Significant at *p* ≤ 0.05

**significant at *p* ≤ 0.01

***significant at *p* ≤ 0.001

Associations between changes in adiposity indicators at the pre-censoring visit and the risk of COVID-19 incidence are presented in Table 3. Every unit reduction in body weight and BMI was significantly associated with lower COVID-19 risk in PREDIMED-Plus participants. However, in the fully adjusted model, only having ≥ 5% reduction in body weight over the follow-up decreased COVID-19 risk on average by 19% (95%CI: 0.04 to 33% reduction) compared to gaining body weight. Losing < 5% of body weight did not appear to have a significant association with the risk of contracting the disease compared to gaining weight (also see Supplementary Table S2- Additional File 4_Table S2). Accordingly, every unit increase in BMI was associated with an increased COVID-19 risk (HR (95%CI): 1.04 (1.003 to 1.08)). Changes in waist circumference and WHtR were not associated with COVID-19 risk. Compared to ABSI measure gains, reductions ≥ 5% were significantly associated with a higher incidence of COVID-19 when fully adjusted (32%, 95%CI: 2 to 72% increase). Supplementary Figure S2 (Additional File 5_Fig. S2) indicates a trend for a higher incidence rate of COVID-19 in participants who had increases in body weight and body mass index compared to those who maintained or had reductions in these measures. This trend was reversed for ABSI and non-prominent for girth measures.

**Table 3:** Changes in adiposity indicators and risk of COVID-19 (HR and 95%CI)

	No. of cases/total	Crude model	Model 1	Model 2
**Body weight change**				
Gain	234/2260	1 (ref)	1 (ref)	1 (ref)
Stable/ < 5% loss	240/2432	0.99 (0.82, 1.18)	0.98 (0.82, 1.18)	0.96 (0.80, 1.16)
> = 5% loss	179/2182	0.78 (0.64, 0.95) *	0.79 (0.65, 0.96) *	0.81 (0.67, 0.996)*
Linear (*per 1 kg increase*)	653/6874	1.01 (1.001, 1.02)*	1.01 (1.00, 1.02)*	1.01 (1.00, 1.03)*
**BMI change**				
Gain	250/2546	1 (ref)	1 (ref)	1 (ref)
Stable/ < 5% loss	279/2269	0.90 (0.75, 1.08)	0.89 (0.74, 1.06)	0.91 (0.76, 1.09)
> = 5% loss	124/2059	0.80 (0.66, 0.97)*	0.80 (0.66, 0.97)*	0.84 (0.69, 1.03)
Linear (*per 1 kg/m*^*2*^* increase*)	653/6874	1.04 (1.003, 1.07)*	1.04 (1.00, 1.07)*	1.04 (1.003, 1.08)*
**Waist circumference change**				
Gain	256/2658	1 (ref)	1 (ref)	1 (ref)
Stable/ < 5% loss	262/2711	0.87 (0.73, 1.04)	1.06 (0.88, 1.26)	0.97 (0.81, 1.16)
≥ 5% loss	135/1370	0.80 (0.65, 0.996) *	0.97 (0.79, 1.20)	0.95 (0.76, 1.18)
Linear (*per 1 cm increase*)	653/6874	1.00 (0.98, 1.01)	1.00 (0.98, 1.01)	1.00 (0.99, 1.01)
**Waist-to-height ratio change (WHtR)**				
Gain	278/2906	1 (ref)	1 (ref)	1 (ref)
Stable/ < 5% loss	244/2527	1.07 (0.91, 1.28)	1.07 (0.90, 1.27)	0.98 (0.82, 1.17)
≥ 5% loss	131/1441	1.00 (0.81, 1.24)	0.99 (0.81, 1.22)	0.98 (0.79, 1.22)
Linear (*per 0.03-unit increase*)	653/6874	0.49 (0.07, 3.38)	0.56 (0.08, 4.01)	1.01 (0.13, 1.73)
**ABSI change**				
Gain	336/3715	1 (ref)	1 (ref)	1 (ref)
Stable/ < 5% loss	244/2523	1.15 (0.97, 1.35)	1.13 (096, 1.34)	1.06 (0.90, 1.26)
≥ 5% loss	73/636	1.37 (1.06, 1.77)*	1.38 (1.07, 1.79)*	1.32 (1.02, 1.72)*
Linear (*per m*^*11/6*^* kg*^*−2/3*^*unit increase*)	653/6874	0.97 (0.95, 0.99)**	0.97 (0.95, 0.99)**	0.97 (0.95, 0.99)**

HR (95% CI) was calculated using Cox proportional regression models. Exposure = changes in adiposity indicators (value at the most recent visit prior to COVID-19 diagnosis or censoring − baseline); outcome: Covid-19 incidence (Y/N)

Gain is defined as any amount of increase from the baseline value, stable/achieving loss signifies maintenance of or less than a 5% reduction from the baseline value. Category ≥ 5% loss = achieving more than a 5% reduction from the baseline value

For modeling the linear association between absolute changes in anthropometric values with COVID-19 risk, respective baseline anthropometric measure was controlled for in the final model. Categorized anthropometric changes were calculated as percentage changes from the baseline and were not adjusted for baseline values

For waist-to-height ratio change, linear association with COVID-19 is calculated per 0.03-unit increase which approximately denotes a 5% increase from the average value for this cohort

The crude model used no adjustments

Model 1: Adjusted for baseline age (years), sex (male/female), education (primary or less/secondary/university), marital status (single or divorced/married/widow(er), recruitment center

Model 2: Additionally, adjusted for baseline smoking status (never/former/current), intervention group, Mediterranean diet adherence score (17-point scale), total physical activity (METs. min./week), alcohol intake (g/d as a quadratic term), and baseline diagnosis of chronic diseases (diabetes, hypertension, hypercholesterolemia (Y/N)), use of ACE-inhibitor at/before pre-censoring visit (Y/N), and having one dose of COVID-19 vaccine at pre-censoring visit (Y/N)

^*^Significant at *p* ≤ 0.05

**significant at *p* ≤ 0.01

***significant at *p* ≤ 0.00

**Fig. 1:**
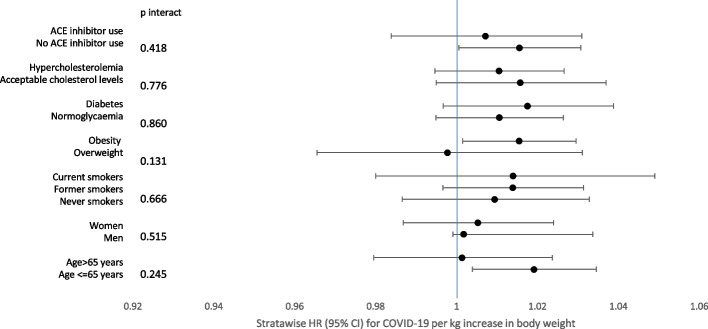
Strata-wise HR (95% CI) for COVID-19 per kg increase in body weight prior to COVID-19 ascertainment

The interaction between potential factors of interest and pre-censoring visit body weight change and strata-wise HR (95% CI) for COVID-19 per kg increase in body weight is shown in Fig. 1. No significant interactions of body weight change were observed with any of the factors tested in their association with COVID-19 risk.

Supplementary analyses that used changes in adiposity parameters that occurred prior to community transmission of COVID-19 in Spain as the exposure did not alter the directionality of the results (Supplementary Table S3, Additional File 6_Table S3). The exclusion of participants who had deceased prior to the emergence of COVID-19 (*n* = 108) for the sensitivity analysis also did not alter the results for associations of COVID-19 risk with baseline adiposity indicators (Supplementary Table S4, Additional File 7_Table S4) or in their changes at the pre-censoring visit (Supplementary Table S5, Additional File 8_Table S5).

## Discussion

We prospectively investigated, the association of baseline adiposity indices and their changes over time with the risk of a SARS-CoV2 infection in the PREDIMED-Plus cohort of older adults with overweight/obesity and metabolic syndrome. Expectedly, several baseline adiposity parameters measured 5–6 years prior to the pandemic were positively associated with COVID-19 risk. In addition, while decreases in body weight and BMI during this period were associated with decreased risk of COVID-19, even when adjusted for baseline values, reductions in girth measures were not associated with significant protective effect against COVID-19.

Previously, several cross-sectional examinations from the early phase of the pandemic found that older adults and those with excessive body weight were typically more vulnerable to severe infection [10–13]. Our study extends these findings by showing that several adiposity parameters in older adults measured 5–6 years prior to the pandemic were also significantly longitudinally associated with increased COVID-19 risk. These associations can be explained by obesity-related metabolic alterations that increase inflammation and result in poor immune response to viruses, including in SARS-CoV2 infections [13, 29]. This suggestion is further supported by a positive association of the total number of leucocytes observed with baseline body weight and body weight gain. It is of interest to note the higher level of total lymphocytes prior to COVID-19 in those who gained body weight (Supplementary Table S1, Additional File 3_Table S1) given a trend for higher lymphocyte level reported in COVID-19-positive patients [28].

Given the health consequences of excess body weight, it is universally recommended that those with overweight/obesity lose weight. However, there is scant information on the effect of the body weight trajectory of individuals in the period prior to COVID-19 on the disease risk, specifically in older adults. The findings from the current analysis suggest the importance of body weight loss in older adults with overweight and obesity, for improving resistance to viral infections such as COVID-19. These results are specifically useful given the expected increase in the emergence and transmission of zoonotic diseases [9] and the globally aging population [16]. The findings importantly show the utility of easily accessible and practical measures such as body weight and BMI in follow-up evaluation among older adults.

The inclusion of ABSI is a unique feature of this analysis that facilitates evaluating baseline or changes in central obesity independent of weight and height parameters (Supplementary Figure S1, Additional File 2_Fig. S1). While waist circumference also evaluates central obesity, it correlates with weight and height and therefore may not be specific for body shape [20]. We found that baseline ABSI was not a predictor of COVID-19 risk in this cohort. This is counter-intuitive given the superiority of ABSI to conventional adiposity parameters such as body weight, BMI, or waist circumference in predicting several mortality in American and European populations [20, 21]. However, both these previous studies included healthy adults aged over 18 years. Given the inclusion criteria of the PREDIMED-PLUS trial, our participants were older and had overweight/obesity and metabolic syndrome. Central obesity which is a hallmark of metabolic syndrome was present in > 99% of PREDIMED-participants. It is possible that older age and predominance of central adiposity diminish the discriminatory ability of baseline ABSI in our participants as demonstrated previously in a cohort of older adults in China [30]. Additionally, larger reductions in ABSI were associated with increased COVID-19 risk in this cohort, although this association was no longer significant in the sensitivity analysis that was restricted to assess changes in adiposity that occurred prior to community transmission of COVID-19. This suggests the potential for residual confounding that could affect the association between changes in ABSI and COVID-19 risk (Supplementary Table S3, Additional File 6_Table S3). Also, baseline but not changes in girth measures were associated with the disease risk unlike body weight and BMI. This could be because body weight and height are less prone to errors as compared to girth measurements making estimates of change less precise [31].

In this context of our study findings, the recent highlight on the need to monitor and investigate weight loss in older adults aged over 65 years by the ASPREE trial is informative [32]. This trial reported that a weight loss of 5% or more and reductions in waist circumference were associated with increased mortality [32]. The authors of the ASPREE trial explained that weight loss commonly precedes a diagnosis of chronic diseases in older adults and that it is associated with a reduced appetite and food intake. The authors have further elaborated on the complex pathways through which appetite suppression in the early stages of chronic disease development is associated with increased inflammation and decreases in muscle mass, muscle strength, and frailty. However, it is important to distinguish two elements of interest between the PREDIMED-Plus study and the ASPREE trial [32], as the health implication of weight loss/gain may be related to age and baseline weight [33]. PREDIMED-Plus participants on average were younger than the ASPREE participants. Secondly, while the ASPREE trial included participants with and without overweight and obesity, the PREDIMED-Plus study used to perform the current analysis is a trial encouraging weight loss and only included older adults with overweight/obesity and metabolic syndrome. While the opportunities for involuntary weight loss are greater in the ASPREE trial, given the nature of the trial, PREDIMED-Plus participants are more likely to have experienced voluntary weight loss. Future evaluations on the health benefits of voluntary weight loss, including reductions in girth in older adults with overweight/obesity, are necessary to tailor recommendations for this age group.

This is one of the first studies to investigate in older adults with overweight/obesity and metabolic syndrome the longitudinal association of baseline anthropometric measures and their changes in 5–6 years prior to the COVID-19 pandemic with the risk of infection. The strength of the analysis lies in its large sample size and documentation of exposures and several confounder variables repeatedly with standardized techniques for a considerable duration before the pandemic. This facilitates the evaluation of the longitudinal association of baseline and changes in adiposity indices with COVID-19 risk while adjusting for several potential confounders, including participant location. Additionally, the duration of follow-up across categories of weight loss is comparable, negating the time-dependent effects of the intervention. Moreover, COVID-19 event adjudication was performed by an independent committee removing any potential bias in the ascertainment of cases. We have also used several adiposity indices including body weight, BMI, waist circumference, and WHtR to define the exposure. This is specifically useful to describe features of general and central obesity and identify differences, if any, in their association with COVID-19 risk.

We also acknowledge the following limitations to this analysis. First, the small number of COVID-19 cases could have lowered the power of the study to identify associations of small magnitude. However, the incidence rate of COVID-19 in this study was similar to the national data reported for the time [34]. Next, since PREDIMED-Plus is currently ongoing, we do not have access to data on the incidence of cancer, diabetes, cardiovascular disease, or illness requiring surgical treatment. The cohort does not have precise measures quantifying body fat%, visceral adiposity, or subcutaneous adiposity. However, we believe while measurements using precision techniques such as dual-energy X-ray absorptiometry (DEXA) are of interest in high-resource research settings, they are not practical for use in primary health care or community settings. Also, weight loss normally accompanies aging [32]. Thus, we cannot ascribe all changes observed in body weight and shape to be voluntary in nature as a consequence of improved lifestyle habits. However, in the sensitivity analysis that confirmed the main findings, we excluded participants who had deceased prior to the onset of the COVID-19 pandemic. This may have offset the above limitation, at least in part, by excluding participants who experienced extreme body weight change due to severe illness. Thirdly, we cannot discount the misclassification of some cases as few participants may have had asymptomatic infections that went undiagnosed. However, we believe this to have been highly unlikely as we scrutinized all medical records during 2020 and 2021 when stringent public health strategies for COVID-19 testing were in place in Spain. Additionally, the supplementary analysis that investigated pre-pandemic anthropometric changes to exclude any reverse causality associated with asymptomatic cases confirmed the original findings. We cannot discount residual confounding given the observational nature of this analysis. Finally, this analysis uses participants included in a clinical trial and therefore caution is necessary while generalizing these results to all older adults or in younger age groups.

## Conclusions

In older adults with overweight/obesity, higher body weight, BMI, waist circumference, and WHtR at baseline, measured 5–6 years prior to the pandemic, were associated with increased COVID-19 risk. Also, every unit reduction in body weight and BMI over this period was associated with decreased COVID-19 risk. Achieving ≥ 5% reductions in body weight loss compared to having gains in these measures was associated with lower COVID-19 incidence even when adjusted for baseline adiposity. In older adults with prior overweight/obesity, achieving clinically significant weight loss may be important to optimize immunity against COVID-19 and potentially other similar infections.

### Supplementary Information

**Additional file 1.** SMethods – [Supplementary Methods].

**Additional file 2:** **Fig. S1.** [Fig S1- Correlations between the baseline adiposity indicators, stratified by sex].

**Additional file 3:** **Table S1.** [Table S1-Participant characteristics according to body weight change at the pre-COVID-19 visit].

**Additional file 4:** **Table S2.** [Table S2- Changes in body weight and risk of COVID-19 (HR & 95%CI)- supplementary analysis (simplified)].

**Additional file 5:** **Fig. S2.** [Fig S2 - Incidence rate of COVID-19 by anthropometric change category].

**Additional file 6:** **Table S3.** [Table S3: Changes in adiposity parameters prior to 8th March 2020 and risk of COVID-19 (HR & 95%CI)-Supplementary analysis].

**Additional file 7:** **Table S4.** [Table S4: Baseline adiposity indicators and the risk of COVID (HR & 95%CI) (Sensitivity analysis)].

**Additional file 8:** **Table S5.** [Table S5: Changes in adiposity indicators and risk of COVID-19 (HR & 95%CI) (Sensitivity analysis)].

**Additional file 9.** STROBE_Checklist.

## Data Availability

The study protocol of PREDIMDED Plus including its statistical analysis plan for the main study has been published earlier [35]. The protocol can also be downloaded from https://www.predimedplus.com/. The datasets generated and analyzed during the current study are not publicly available due to data regulations and ethical reasons. However, collaboration for data analyses can be requested by sending a letter to the PREDIMED-Plus Steering Committee (predimed_plus_scommittee@googlegroups.com). The request will then be passed to all the members of the PREDIMED-Plus Steering Committee for deliberation.

## Abbreviations

ABSI: A body shape index
ACE: Angiotensin-converting enzyme
BMI: Body mass index
CI: Confidence interval
COVID-19: Coronavirus disease-2019
HR: Hazard ratio
IQR: Interquartile range
SARS-CoV2: Severe acute respiratory syndrome coronavirus-2
WHtR: Waist-to-height ratio
