# Supplementary material for: Association of adiposity and its changes over time with COVID-19 risk in older adults with overweight/obesity and metabolic syndrome: a longitudinal evaluation in the PREDIMED-Plus cohort
Source: BMC Med. 2023 Oct 13;21:390. doi: 10.1186/s12916-023-03079-z (PMC10576302; doi:10.1186/s12916-023-03079-z)
Supplement: Supplementary file 1 — Additional file 1. SMethods – [Supplementary Methods]. [file 12916_2023_3079_MOESM1_ESM.docx]

# Supplementary Material

## Supplementary Methods

Ascertainment of COVID-19 event

A COVID-19 event was ascertained as follows: a) Confirmed COVID-19 was defined as a positive response to any one of the following tests: PCR SARS-CoV-2 (acute infection), Ag SARS-CoV-2 (acute infection), Ab SARS-CoV-2, total or IgG (past infection) and b) Probable COVID-19 infection was defined as Ab SARS-CoV-2, IgM + without a subsequent increase in IgG (IgG -) or a clinical presentation compatible diagnosis of COVID-19 by an attending physician treating COVID-19, without analytical testing at or after the acute moment. Participants who had a confirmed (n=652)/probable (n=1) COVID-19 diagnosis were considered COVID-19 incident cases (n= 653), and others as COVID-19 non-incident (n=6,221) in this analysis.
