# Supplementary figures and images for: Association of adiposity and its changes over time with COVID-19 risk in older adults with overweight/obesity and metabolic syndrome: a longitudinal evaluation in the PREDIMED-Plus cohort
Source: BMC Med. 2023 Oct 13;21:390. doi: 10.1186/s12916-023-03079-z (PMC10576302; doi:10.1186/s12916-023-03079-z)

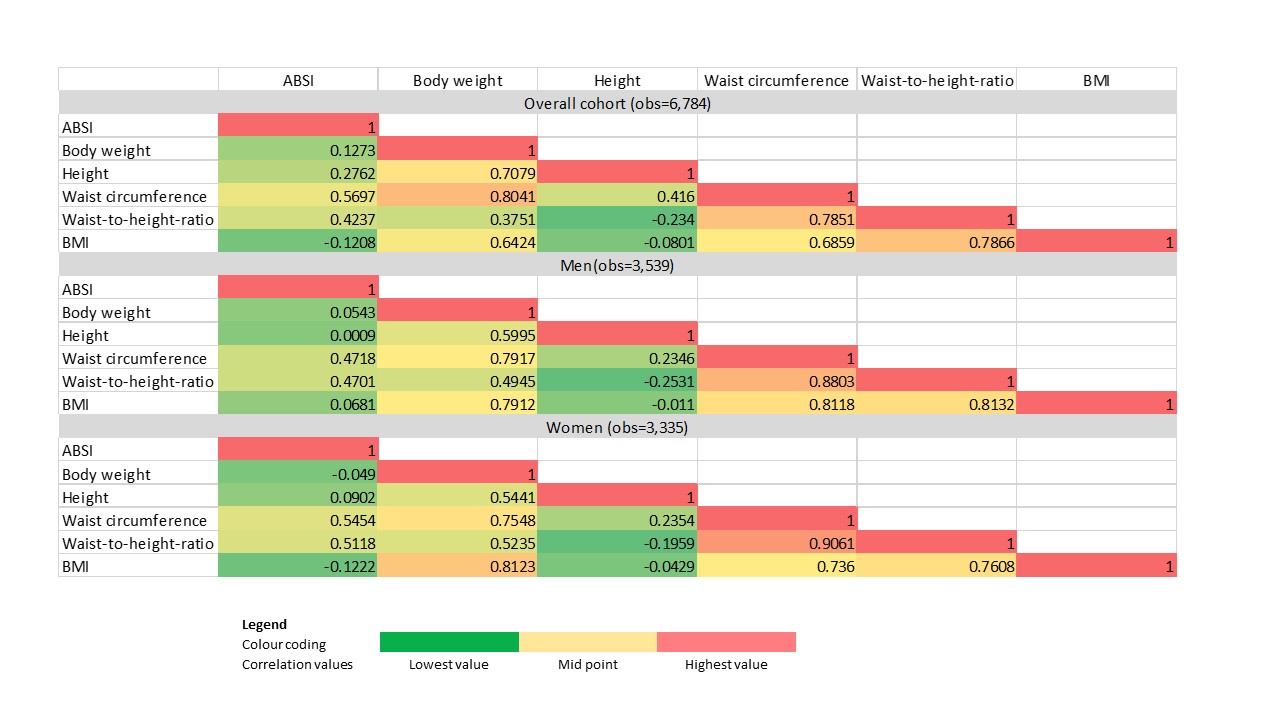

Supplement: Supplementary file 2 — Additional file 2: Fig. S1. [Fig S1- Correlations between the baseline adiposity indicators, stratified by sex]. [file 12916_2023_3079_MOESM2_ESM.jpg]

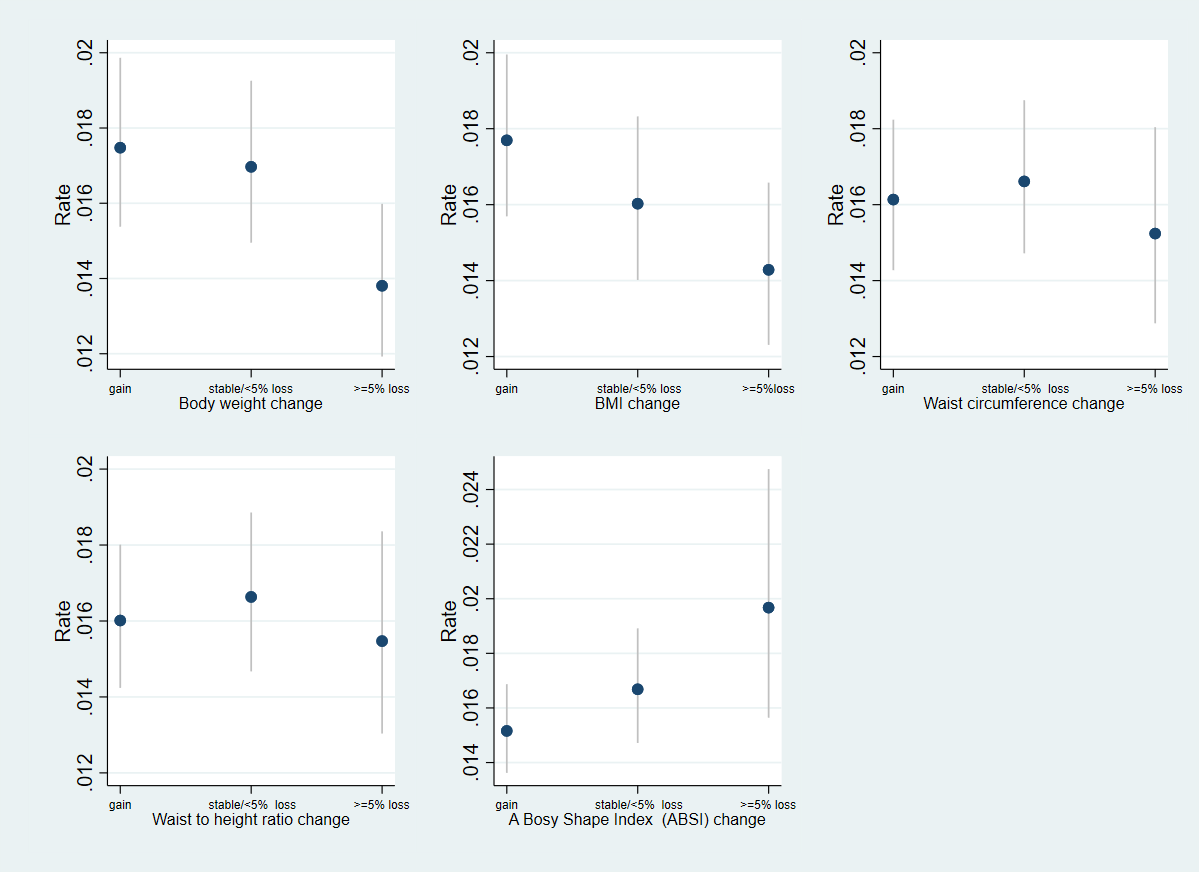

Supplement: Supplementary file 5 — Additional file 5: Fig. S2. [Fig S2 - Incidence rate of COVID-19 by anthropometric change category]. [file 12916_2023_3079_MOESM5_ESM.png]
