# Supplementary material for: Association of adiposity and its changes over time with COVID-19 risk in older adults with overweight/obesity and metabolic syndrome: a longitudinal evaluation in the PREDIMED-Plus cohort
Source: BMC Med. 2023 Oct 13;21:390. doi: 10.1186/s12916-023-03079-z (PMC10576302; doi:10.1186/s12916-023-03079-z)
Supplement: Supplementary file 3 — Additional file 3: Table S1. [Table S1-Participant characteristics according to body weight change at the pre-COVID-19 visit]. [file 12916_2023_3079_MOESM3_ESM.docx]

Supplementary Table S1. Participant characteristics according to body weight change at the pre-COVID-19 visit

|  | Body weight change category^t^ | | | P-value^a^ |
| --- | --- | --- | --- | --- |
|  | Weight gain | Weight stable/<5% weight loss | Weight loss ≥ 5% |  |
|  | (n=2,260) | (n=2,432) | (n=2,182) |  |
| Sociodemographic data |  |  |  |  |
| Age years Mean (SD) ^b^ | 64.7 (4.9) | 65.0 (5.0) | 65.2 (4.9) | 0.008 |
| Men, n (%) | 1,100 (48.7) | 1,297 (53.3) | 1,142 (52.3) | 0.004 |
| Education level, n (%) ^b^ |  |  |  | 0.625 |
| Primary school or less | 1,129(50.0) | 1,168 (48.0) | 1,065 (48.9) |  |
| High school or equivalent | 648 (28.7) | 702(28.9) | 636 (29.0) |  |
| University | 483 (21.4) | 562 (23.1) | 481 (22.2) |  |
| Civil status, n (%)^b^ |  |  |  | 0.029 |
| Single or divorced | 328 (14.6) | 312 (12.9) | 257 (11.8) |  |
| Married | 1,683 (74.7) | 1,845 (76.2) | 1,706 (78.6) |  |
| Widow/Widower | 241 (10.7) | 266 (11.0) | 209 (9.6) |  |
| Intervention group (Allocation to Group B) | 818 (36.2) | 1,228 (50.5) | 1,360 (62.3) | <0.001 |
| Lifestyle habits |  |  |  |  |
| Smoking habit, n (%) ^b^ |  |  |  | 0.021 |
| Never smoker | 974 (43.1) | 1,072 (44.1) | 988 (45.2) |  |
| Former smoker | 970 (42.9) | 1,092 (44.9) | 921 (42.2) |  |
| Current smoker | 316 (14.0) | 268 (11.0) | 273 (12.5) |  |
| Study mean 17-item MedDiet score_1_ ^b#^ | 9.0 (3.0) | 8.0 (3.0) | 8.0 (4.0) | 0.004 |
| Total physical activity, METs. min./week ^b #^ | 1928  (2624) | 1904  (2580) | 1734  (2739) | 0.011 |
| Alcohol consumption, g/d ^b #^ | 4.5 (14.0) | 5.1 (14.1) | 5.1 (14.0) | 0.238 |
| Anthropometry and clinical data |  |  |  |  |
| Weight (kg) ^#b^ | 84.7 (17.3) | 85.5 (18.0) | 86.5(18.2) | 0.001 |
| Height (cm) ^#b^ | 162 (14.0) | 163 (14.0) | 163 (14.5) | 0.001 |
| BMI, kg/m^2 #b^ | 32.0(5.1) | 32.0 (5.0) | 32.5(5.2) | <0.001 |
| Waist circumference (cm) ^#b^ | 106.0 (12.9) | 106.9(12.8) | 108.0 (13.0) | <0.001 |
| Waist-to-height ratio^# b^ | 0.65(0.08) | 0.65(0.08) | 0.66(0.08) | <0.001 |
| ABSI^#b^ | 82.8 (5.7) | 83.0 (5.6) | 83.2 (5.6) | 0.045 |
| Obesity; BMI≥30, n (%) ^b^ | 1,623 (71.8) | 1,763 (72.5) | 1,660 (76.1) | 0.003 |
| Diabetes, n (%) ^b^ | 564 (25.0) | 752 (30.9) | 805 (36.9) | <0.001 |
| Hypercholesterolemia, n (%) ^b^ | 1,577 (69.8) | 1,707 (70.2) | 1,529 (70.1) | 0.952 |
| Hypertension, n (%) ^b^ | 1,871 (82.8) | 2,049 (84.3) | 1,838 (84.3) | 0.307 |
| Total, leucocytes (×10^e9^/L)^# b^ | 6.63 (2.21) | 6.60 (2.12) | 6.46 (2.25) | 0.008 |
| Lymphocytes (×10^e9^/L)^# b^ | 2.08 (0.94) | 2.04 (0.93) | 1.98 (0.90) | <0.001 |
| Platelets (×10^e9^/L)^# b^ | 225 (73) | 222(74) | 219(73) | 0.042 |
| Hemoglobin (g/dL) | 14.4 (1.8) | 14.5 (1.9) | 14.3 (2.0) | 0.013 |
| Use of ACE inhibitors, n (%)^t^ | 670 (29.7) | 799 (32.8) | 696 (31.9) | 0.054 |
| Received at least 1 dose of vaccine, n (%)^t^ | 1,166 (51.6) | 1,177 (48.4) | 1,160 (53.2) | 0.004 |
| COVID-19 incident cases, n (%) | 234 (10.4) | 240 (9.9) | 179 (8.2) | 0.037 |
| Time in trial at pre-COVID-19 visit (years) ^#^ | 4.0 (1.2) | 4.0 (1.2) | 4.1 (1.2) | 0.205 |
| Survival time (years) ^#^ | 5.8 (1.3) | 5.7 (1.2) | 5.8 (1.3) | 0.013 |

Abbreviations: ABSI, A Body Shape Index; ACE, Angiotensin-Converting-Enzyme; BMI, body mass index; COVID-19, coronavirus disease 2019; MedDiet, Mediterranean diet

_1_Notes on scales: Possible MedDiet scores range between 0 and 17. Higher MedDiet scores represent higher adherence to the Mediterranean diet.

A Body Shape Index (ABSI was calculated as waist circumference X weight^-2/3^ X height^5/6^. ABSI was multiplied by 1000 to facilitate interpretation.

Data are n (%) for categorical variables. ^#^Unless specified, quantitative data are presented as median (IQR)). b Data from study baseline; ^t^Data at the time of PRE-COVID visit.

^C^ Body weight change refers to the difference in body weight between the value at the last visit prior to censoring and the baseline value. Weight gain is defined as any amount of weight gain, weight stable refers to achieving 0-5% weight loss in relation to initial body weight, and weight loss refers to achieving >=5%.

^a^P-values for comparisons between groups were tested using the Kruskal Wallis test (owing to the skewed nature of the distribution) or χ2, as appropriate.

Age, sex, education, intervention group, recruitment centre, baseline physical activity anthropometry, prevalence of diabetes, hypertension and hypercholesterolemia had no missing data for this analysis. Baseline smoking status: 28/6,874 (0.4% missing data). Marital status at: 27/6874 (0.4%) missing data. Missing data for these two variables were replaced with the mode of the variable for the cohort. Alcohol consumption at baseline: 36/6,784 (0.5%) missing data. Missing data was replaced with cohort mean consumption by gender (men= 17.47276; women= 4.599879 g/day). Mediterranean diet adherence: 5/6,784 missing (<0.1%) missing data. Missing data was replaced with cohort mean.
