# Supplementary material for: Association of adiposity and its changes over time with COVID-19 risk in older adults with overweight/obesity and metabolic syndrome: a longitudinal evaluation in the PREDIMED-Plus cohort
Source: BMC Med. 2023 Oct 13;21:390. doi: 10.1186/s12916-023-03079-z (PMC10576302; doi:10.1186/s12916-023-03079-z)
Supplement: Supplementary file 4 — Additional file 4: Table S2. [Table S2- Changes in body weight and risk of COVID-19 (HR & 95%CI)- supplementary analysis (simplified)]. [file 12916_2023_3079_MOESM4_ESM.docx]

Supplementary Table S2: Changes in body weight and risk of COVID-19 (HR & 95%CI)- supplementary analysis (simplified)

| Weight change status | No of cases/total | Crude Model | Model 1 | Model 2 | Model 3 |
| --- | --- | --- | --- | --- | --- |
| Weight gain | 234/2,260 | 1 (ref) | 1 (ref) | 1 (ref) | 1 (ref) |
| Stable weight /weight loss | 419/4,614 | 0.89 (0.76, 01.04) | 0.89 (0.76, 1.05) | 0.90 (0.76, 1.06) | 0.91 (0.77, 1.08) |

HR (95% CI) was calculated using Cox Proportional regression models. Exposure= Pre-COVID-19 body weight change; outcome: Covid-19 incidence (Y/N).

Body weight change refers to the difference in body weight between the value at the last visit prior to censoring and the baseline value. Weight gain is defined as any amount of weight gain, and stable weight/weight loss is defined as achieving 0 to any amount of weight loss.

The crude model used no adjustments.

Model 1: Adjusted for baseline age (y), sex (Male/Female), education (Primary or less/Secondary/University), marital status (Single or divorced/Married/Widow(er), recruitment center.

Model 2: Additionally, adjusted for baseline smoking status (Never/former/current), Mediterranean diet adherence score (17-point scale), total physical activity (METs.min./week), alcohol intake (g/d as a quadratic term), and previous diagnosis of chronic diseases (diabetes, hypertension, hypercholesterolemia (Y/N)), use of ace-inhibitor at/prior to pre-censoring visit (Y/N), and having one dose of COVID-19 vaccine at the time of censoring (Y/N).

Model 3: Model 2 additionally for total number of leucocytes(×10^e9^/L) data at the last available visit prior to COVID-19

*Significant at p≤ 0.05, ** Significant at p≤ 0.01, *** Significant at p≤ 0.001
