# Supplementary material for: Association of adiposity and its changes over time with COVID-19 risk in older adults with overweight/obesity and metabolic syndrome: a longitudinal evaluation in the PREDIMED-Plus cohort
Source: BMC Med. 2023 Oct 13;21:390. doi: 10.1186/s12916-023-03079-z (PMC10576302; doi:10.1186/s12916-023-03079-z)
Supplement: Supplementary file 6 — Additional file 6: Table S3. [Table S3: Changes in adiposity parameters prior to 8th March 2020 and risk of COVID-19 (HR & 95%CI)-Supplementary analysis]. [file 12916_2023_3079_MOESM6_ESM.docx]

Supplementary Table S3: Changes in adiposity parameters prior to 8^th^ March 2020† and risk of COVID-19 (HR & 95%CI)-Supplementary analysis

|  | No. of cases/total | Crude Model | Model 1 | Model 2 |
| --- | --- | --- | --- | --- |
| Body weight change |  |  |  |  |
| Gain | 236/2,182 | 1 (ref) | 1 (ref) | 1 (ref) |
| Stable /<5% loss | 238/ 2,732 | 0.84 (0.70, 1.00) | 0.84 (0.70, 1.00) | 0.85 (0.70, 1.02) |
| >=5% loss | 179/ 1,960 | 0.85 (0.70, 1.03) | 0.86 (0.71, 1.04) | 0.87 (0.71,1.06) |
| Linear (*per 1 kg increase*) | 653/6,874 | 1.01 (1.00,1.02) | 1.01 (0.99,1.02) | 1.01 (1.00,1.03) |
| BMI change |  |  |  |  |
| Gain | 280/2,508 | 1 (ref) | 1 (ref) | 1 (ref) |
| Stable /<5% loss | 206/2,498 | 0.75 (0.62,0.89)** | 0.75 (0.62,0.89)** | 0.75 (0.63,0.90)** |
| >=5% loss | 167/1,868 | 0.82 (0.68,0.99)* | 0.82 (0.68,0.998)* | 0.82 (0.68,1.01) |
| Linear (*per 1 kg/m^2^ increase*) | 653/6,874 | 1.03 (0.99,1.07) | 1.03(0.99,1.07) | 1.03(0.99,1.07) |
| Waist circumference change |  |  |  |  |
| Gain | 233/ 2,325 | 1 (ref) | 1 (ref) | 1 (ref) |
| Stable /<5% loss | 275/ 2,970 | 0.99 (0.83,1.18) | 0.98 (0.82,1.16) | 1.00 (0.82,1.18) |
| >=5% loss | 145/1,579 | 0.97 (0.79,1.20) | 0.96 (0.78,1.19) | 0.99 (0.79,1.21) |
| Linear (*per 1 cm increase*) | 653/6,874 | 1.02 (0.99,1.01) | 1.00 (0.99,1.01) | 1.00 (0.99,1.01) |
| Waist-to-height ratio change (WHtR) |  |  |  |  |
| Gain | 253/2,552 | 1 (ref) | 1 (ref) | 1 (ref) |
| Stable /<5% loss | 260/ 2,798 | 1.01 (0.85,1.20) | 1.00 (0.84,1.19) | 1.01 (0.85,1.20) |
| >=5% loss | 140/ 1,524 | 0.98 (0.80,1.21) | 0.97 (0.79,1.20) | 0.98 (0.69,1.22) |
| Linear (*per 0.03-unit increase*) | 653/6,874 | 1.02 (0.12, 8.25) | 1.07 (0.13,9.07) | 1.43 (0.16,12.3) |
| ABSI Change |  |  |  |  |
| Gain | 322/3,243 | 1 (ref) | 1 (ref) | 1 (ref) |
| Stable /<5% loss | 257/2,956 | 0.93 (0.80, 1.10) | 0.92 (0.78, 1.09) | 0.93(0.78, 1.09) |
| >=5% loss | 74/675 | 1.17 (0.91, 1.51) | 1.17 (0.91, 1.51) | 1.17 (0.91, 1.51) |
| Linear (*per unit increase*) | 653/6,874 | 1.00 (0.99,1.00) | 1.00 (0.99, 1.00) | 1.00 (0.99, 1.00) |

Table Legend: ^#^HR (95% CI) was calculated using Cox Proportional regression models. Exposure= changes in adiposity indicators (†value at the most recent visit prior to 8^th^ March 2020, the date of documented community transmission of COVID-19 in Spain- baseline); outcome: Covid-19 incidence (Y/N).

Gain is defined as any amount of increase from the baseline value, Stable/achieving loss signifies maintenance of or less than a 5% reduction from the baseline value. >=5% loss = achieving more than a 5% reduction from the baseline value.

For modelling the linear association between absolute changes in anthropometric values with COVID-19 risk, baseline anthropometric measure was controlled for in the final model. Categorized anthropometric changes were calculated as percentage changes from the baseline and were not adjusted for baseline values.

For waist-to-height ratio change, linear association with COVID-19 is calculated per 0.03-unit increase which approximately denotes a 5% increase from the average value for this cohort.

The crude model used no adjustments.

Model 1: Adjusted for Baseline age (y), sex (Male/Female), education (Primary or less/Secondary/University), marital status (Single or divorced/Married/Widow(er), recruitment center.

Model 2: Additionally, adjusted for baseline smoking status (Never/former/current), Mediterranean diet adherence score (17-point scale), total physical activity (METs.min./week), alcohol intake (g/d as a quadratic term), and previous diagnosis of chronic diseases (diabetes, hypertension, hypercholesterolemia (Y/N)), use of ace-inhibitor at/prior to pre-censoring visit (Y/N), and having one dose of COVID-19 vaccine at the time of censoring (Y/N).

*Significant at p≤ 0.05, ** Significant at p≤ 0.01, *** Significant at p≤ 0.001
