# Supplementary material for: Association of adiposity and its changes over time with COVID-19 risk in older adults with overweight/obesity and metabolic syndrome: a longitudinal evaluation in the PREDIMED-Plus cohort
Source: BMC Med. 2023 Oct 13;21:390. doi: 10.1186/s12916-023-03079-z (PMC10576302; doi:10.1186/s12916-023-03079-z)
Supplement: Supplementary file 7 — Additional file 7: Table S4. [Table S4: Baseline adiposity indicators and the risk of COVID (HR & 95%CI) (Sensitivity analysis)]. [file 12916_2023_3079_MOESM7_ESM.docx]

Supplementary Table S4: Baseline adiposity indicators and the risk of COVID (HR & 95%CI) (Sensitivity analysis)#

|  | No of cases/total | Crude Model | Model 1 | Model 2 |
| --- | --- | --- | --- | --- |
| Body weight |  |  |  |  |
| Tertile 1 | 169/2,265 | 1 (ref) | 1 (ref) | 1 (ref) |
| Tertile 2 | 222/2,266 | 1.34 (1.09,1.63) ** | 1.27 (1.03, 1.57) * | 1.26 (1.02,1.55) * |
| Tertile 3 | 262/2235 | 1.62(1.34, 1.96) *** | 1.48 (1.19, 1.85) *** | 1.47 (1.17,1.84) *** |
| Linear (*per 1 kg increase*) | 653/6766 | 1.01 (1.01,1.02) *** | 1.01 (1.005,1.02) *** | 1.01 (1.004,1.02) *** |
|  |  |  |  |  |
| Body mass index (BMI) |  |  |  |  |
| category: overweight | 145/1,803 | 1 (ref) | 1 (ref) | 1 (ref) |
| category: obesity | 508/4,963 | 1.28 (1.06, 1.54) ** | 1.29 (1.07, 1.56) ** | 1.27 (1.05, 1.54) * |
| BMI linear (*per 1 kg/m^2^ increase*) | 653/6766 | 1.04 (1.02, 1.06) *** | 1.04 (1.02, 1.06) *** | 1.04 (1.02, 1.06) *** |
|  |  |  |  |  |
| Waist circumference |  |  |  |  |
| Tertile 1 | 185/2,339 | 1 (ref) | 1 (ref) | 1 (ref) |
| Tertile 2 | 229/2,175 | 1.33 (1.09,1.62) ** | 1.28 (1.05,1.57) * | 1.21 (0.99,1.49) |
| Tertile 3 | 239/2,252 | 1.37(1.13,1.67) *** | 1.29 (1.05,1.59) * | 1.22(0.99, 1.51) |
| waist circumference linear (*per 1 cm increase*) | 653/6766 | 1.02 (1.01,1.02) *** | 1.01 (1.004,1.02) ** | 1.01 (1.003,1.02) * |
|  |  |  |  |  |
| Waist-to-height ratio (WHtR) |  |  |  |  |
| Tertile 1 | 224/2,258 | 1 (ref) | 1 (ref) | 1 (ref) |
| Tertile 2 | 176/2,253 | 0.78(0.64,0.95) * | 0.82 (0.67, 0.996) * | 0.80 (0.65,0.98) * |
| Tertile 3 | 253/2,255 | 1.16 (0.97,1.39) | 1.24 (1.03, 1.49) * | 1.21 (1.001,1.46) * |
| WHtR linear (*per 0.03-unit increase*) | 653/6766 | 1.04 (1.00,1.08) | 1.06 (1.01,1.10) ** | 1.05 (1.01, 1.10) * |
|  |  |  |  |  |
| ABSI |  |  |  |  |
| Tertile 1 | 224/2,268 | 1 (ref) | 1 (ref) | 1 (ref) |
| Tertile 2 | 220/2,256 | 0.99 (0.82, 1.19) | 0.94 (0.77, 1.13) | 0.94 (0.77, 1.13) |
| Tertile 3 | 209/2,242 | 0.95 (0.79, 1.15) | 0.91 (0.74, 1.12) | 0.91 (0.71, 1.08) |
| Linear (*per m^11/6^ kg^-2/3^unit increase*) | 653/6,766 | 1.00 (0.98, 1.02) | 1.00 (0.98, 1.02) | 0.99 (0.97, 1.01) |

#Sensitivity analysis excluded participants (n=108) who had deceased before 30, November 2019, thus excluding those with no possibility of being diagnosed with COVID-19.

^#^HR (95% CI) was calculated using Cox Proportional regression models. Exposure= baseline anthropometric data; outcome: Covid-19 incidence (Y/N).

For waist-to-height ratio change, linear association with COVID-19 is calculated per 0.03-unit increase which approximately denotes a 5% increase from the average value for this cohort.

A Body Shape Index (ABSI was calculated as waist circumference X weight^-2/3^ X height^5/6^. ABSI was multiplied by 1000 to facilitate interpretation.

The crude model used no adjustments.

Model 1: Adjusted for baseline age (y), sex (Male/Female), education (Primary or less/ Secondary/University), marital status (Single or divorced/Married/Widow(er), and recruitment center

Model 2: Additionally, adjusted for baseline smoking status (Never/former/current), mean Mediterranean diet adherence score (17-point scale), mean total physical activity (METs.min./week), alcohol intake at pre-censoring visit (g/d as a quadratic term), and baseline diagnosis of chronic diseases (diabetes, hypertension, hypercholesterolemia (Y/N)), use of ace-inhibitor at/prior to pre-censoring visit (Y/N) and having one dose of COVID-19 vaccine at the time of censoring (Y/N).

*Significant at p≤ 0.05, ** Significant at p≤ 0.01, *** Significant at p≤ 0.001
