# Supplementary material for: Association of adiposity and its changes over time with COVID-19 risk in older adults with overweight/obesity and metabolic syndrome: a longitudinal evaluation in the PREDIMED-Plus cohort
Source: BMC Med. 2023 Oct 13;21:390. doi: 10.1186/s12916-023-03079-z (PMC10576302; doi:10.1186/s12916-023-03079-z)
Supplement: Supplementary file 8 — Additional file 8: Table S5. [Table S5: Changes in adiposity indicators and risk of COVID-19 (HR & 95%CI) (Sensitivity analysis)]. [file 12916_2023_3079_MOESM8_ESM.docx]

Supplementary Table S5: Changes in adiposity indicators and risk of COVID-19 (HR & 95%CI) (Sensitivity analysis)#

|  | No. of cases/total | Crude Model | Model 1 | Model 2 |
| --- | --- | --- | --- | --- |
| Body weight change |  |  |  |  |
| Weight gain | 234/2,245 | 1 (ref) | 1 (ref) | 1 (ref) |
| Stable /<5% loss | 240/2,370 | 0.99 (0.82, 1.18) | 0.99 (0.82, 1.18) | 0.97 (0.80, 1.16) |
| >=5% loss | 179/2,151 | 0.78 (0.64, 0.95) * | 0.79 (0.65, 0.96) * | 0.81 (0.67, 0.999) * |
| Linear (*per 1 kg increase*) | 653/6,766 | 1.01 (1.00,1.02)* | 1.01 (1.00,1.02)* | 1.01 (0.999,1.03) |
| BMI change |  |  |  |  |
| Gain | 266/2,517 | 1 (ref) | 1 (ref) | 1 (ref) |
| Stable /<5% loss | 214/2,234 | 0.91(0.76,1.09) | 0.89(0.75,1.07) | 0.91 (0.76,1.10) |
| >=5% loss | 173/2,015 | 0.81(0.67,0.98)* | 0.80(0.66,0.97)* | 0.84 (0.69,1.03) |
| Linear (*per 1 kg/m^2^ increase*) | 653/6,766 | 1.04 (1.003,1.07)* | 1.04 (1.004,1.07)* | 1.04 (1.004,1.08)* |
| Waist circumference change |  |  |  |  |
| Gain | 256/ 2,639 | 1 (ref) | 1 (ref) | 1 (ref) |
| Stable /<5% loss | 262/ 2,640 | 1.06(0.89, 1.26) | 1.06(0.89, 1.26) | 0.97(0.81, 1.16) |
| >=5% loss | 135/1,487 | 0.97(0.79,1.20) | 0.97(0.79,1.20) | 0.95(0.76,1.18) |
| Linear *(per 1 cm increase)* | 653/6,766 | 1.04 (0.98,1.01) | 1.00 (0.98,1.01) | 1.00 (0.99,1.01) |
| Waist-to-height ratio change |  |  |  |  |
| Gain | 278/2,884 | 1 (ref) | 1 (ref) | 1 (ref) |
| Stable /<5% loss | 244/2,458 | 1.08(0.91,1.28) | 1.07(0.90,1.28) | 1.00(0.83,1.18) |
| >=5% loss | 131/1,424 | 1.00(0.81,1.23) | 1.00(0.81,1.23) | 0.99(0.80,1.22) |
| Linear (*per 0.03-unit increase*) | 653/6,766 | 0.49(0.07,3.29) | 0.51(0.07,3.66) | 0.84(0.11,6.46) |
| ABSI Change |  |  |  |  |
| Gain | 336/3677 | 1 (ref) | 1 (ref) | 1 (ref) |
| Stable /<5% loss | 244/2458 | 1.15 (0.97, 1.35) | 1.13 (0.96, 1.34) | 1.06 (0.90, 1.26) |
| >=5% loss | 73/631 | 1.37 (1.06, 1.77)* | 1.34 (1.07, 1.79)* | 1.33 (1.02, 1.73)* |
| Linear (*per m^11/6^ kg^-2/3^unit increase*) | 653/6,766 | 0.97 (0.95, 0.99)** | 0.97 (0.95, 0.99)** | 0.98 (0.96, 0.999)* |

#Sensitivity analysis excluded participants (n=108) who had deceased before 30, November 2019, thus excluding those with no possibility of being diagnosed with COVID-19.

Table Legend: ^#^HR (95% CI) was calculated using Cox Proportional regression models. Exposure= changes in adiposity indicators (value at the most recent visit prior to COVID-19 diagnosis or censoring - baseline); outcome: Covid-19 incidence (Y/N).

Gain is defined as any amount of increase from the baseline value, Stable/achieving loss signifies maintenance of or less than a 5% reduction from the baseline value. >=5% loss = achieving more than a 5% reduction from the baseline value.

For modelling the linear association between absolute changes in anthropometric values with COVID-19 risk, baseline anthropometric measure was controlled for in the final model. Categorized anthropometric changes were calculated as percentage changes from the baseline and were not adjusted for baseline values.

For waist-to-height ratio change, linear association with COVID-19 is calculated per 0.03-unit increase which approximately denotes a 5% increase from the average value for this cohort.

The crude model used no adjustments.

Model 1: Adjusted for baseline age (y), sex (Male/Female), education (Primary or less/ Secondary/University), marital status (Single or divorced/ Married/ Widow(er), and recruitment center

Model 2: Additionally, adjusted for baseline smoking status (Never/former/current), Mediterranean diet adherence score (17-point scale), total physical activity (METs.min./week), alcohol intake (g/d as a quadratic term), and previous diagnosis of chronic diseases (diabetes, hypertension, hypercholesterolemia (Y/N)), use of ace-inhibitor at/prior to pre-censoring visit (Y/N), and having one dose of COVID-19 vaccine at the time of censoring (Y/N).

*Significant at p≤ 0.05, ** Significant at p≤ 0.01, *** Significant at p≤ 0.001
